# Law Enforcement Drug Seizures and Opioid-Involved Overdose Mortality

**DOI:** 10.1001/jamanetworkopen.2025.1158

**Published:** 2025-03-19

**Authors:** Alex H. Kral, Jamie L. Humphrey, Clyde Schwab, Barrot H. Lambdin, Bradley Ray

**Affiliations:** 1 RTI International, Research Triangle Park, North Carolina; 2 Department of Epidemiology and Biostatistics, University of California, San Francisco; 3 Department of Global Health, University of Washington, Seattle

## Abstract

**Importance:**

Opioid-involved overdose mortality has been on the rise for 2 decades in the US, exacerbated by an unregulated drug supply that is unpredictable and has increasingly contained highly potent fentanyl analogs starting a decade ago.

**Objective:**

To determine whether there is a geospatial association between law enforcement drug seizures and opioid-involved overdose mortality in San Francisco.

**Design, Setting, and Participants:**

This cross-sectional study used location- and time-stamped overdose mortality data from the Office of the Chief Medical Examiner and publicly available crime data from the San Francisco Police Department between 2020 and 2023 to assess whether location and time of law enforcement drug seizures were associated with subsequent opioid-involved overdose mortality. Data were analyzed from January 2020 to September 2023.

**Exposures:**

Time-stamped locations of law enforcement drug seizures involving a drug distribution charge.

**Main Outcomes and Measures:**

The primary outcomes were the time and location of (1) overdose mortality involving any opioid and (2) overdose mortality involving fentanyl or any fentanyl analog. The relative risk (RR) and 95% CIs for endemic and epidemic factors were calculated.

**Results:**

There were 2653 drug seizure crime events that involved any drug distribution charge and 1833 overdose deaths that tested positive for any opioid or synthetic opioid, including heroin and fentanyl analogs. Within the surrounding 100 meters, law enforcement drug seizures were associated with increase risk of fatal opioid-involved overdoses the day following the drug seizure event (RR, 1.74; 95% CI, 1.06-2.83; *P* = .03) and elevated risk persisted for 7 days (2 days: RR, 1.55; 95% CI, 1.09-2.21; *P* = .02; 3 days: RR, 1.45; 95% CI, 1.08-1.93; *P* = .01; 7 days: RR, 1.27; 95% CI, 1.11-1.46; *P* = .001). Similar statistically significant spatiotemporal patterns were observed in the 250- and 500-meter spatial bandwidths. Within each space-time kernel, the strength of the association, all of which were statistically significant, dissipated the further away in time and distance from the law enforcement drug seizure event.

**Conclusions and Relevance:**

The findings of this cross-sectional study suggest that the enforcement of drug distribution laws to increase public safety for residents in San Francisco may be having an unintended negative consequence of increasing opioid overdose mortality. To reduce overdose mortality, it may be better to focus on evidence-based health policies and interventions.

## Key Points

**Question:**

Is there a geospatial association between opioid-involved overdose mortality and law enforcement drug seizures in San Francisco?

**Findings:**

This cross-sectional study included 2653 drug seizure crime events. Within the surrounding 100, 250, and 500 meters, drug seizures were associated with a statistically significant increase in the relative risk for fatal opioid overdoses 1, 2, 3, and 7 days following law enforcement drug seizure events.

**Meaning:**

These findings suggest that the enforcement of drug distribution laws to increase public safety for residents in San Francisco may be having an unintended negative consequence of increasing opioid overdose mortality.

## Teaser

This cross-sectional study examines the geospatial association between law enforcement drug seizures and opioid-involved overdose mortality in San Francisco, California, between 2020 and 2023.

## Introduction

Opioid-involved overdose mortality has been on the rise for 2 decades in the US, exacerbated by an unregulated drug supply that is unpredictable and has increasingly contained highly potent fentanyl analogs starting in 2014. Provisional estimates from the US Centers for Disease Control and Prevention indicate there were 107 543 overdose deaths in 2023, in which 74 702 involved opioids or synthetic opioids like fentanyl.^1^ Illicitly manufactured fentanyl entered the unregulated drug supply on the east coast in 2013, and the west coast since 2019, which consequently experienced the largest increases in opioid-involved overdose mortality from 2020 to 2023.^2,3,4^

Two main drivers of opioid-involved overdoses are using drugs procured from an unregulated drug supply and temporary interruptions of opioid use for people who are dependent upon opioids. The unregulated drug supply leads to overdose risk because the person procuring the drugs does not know the complete content or potency of the drug solution they procure and ingest. As such, people using unregulated drugs may inadvertently be using different drugs than they intended, leading to overdose. Temporary interruptions of opioid use also present an overdose risk for people who are dependent,^5^ as most use them regularly, often daily,^6^ or they risk severe withdrawal symptoms.^7^ Once they are experiencing withdrawal symptoms, many will go to great lengths to urgently procure and use opioids to stave off these symptoms.^8,9^

The sale, possession, and use of opioids that were not prescribed to the person using the opioids has been illegal in the US since the Controlled Substances Act of 1970.^10^ Federal, state, and local law enforcement regularly arrest people who sell opioids illicitly in communities, leaving those dependent on them unable to have a reliable source. Given the unpredictability of the drugs obtained in the unregulated drug market, people pushed to obtain potentially more potent drug solutions elsewhere face higher overdose risk.^11^ Recent research in Indianapolis, Indiana, has indicated spatiotemporal associations between law enforcement efforts to disrupt illicit opioid markets and fatal overdose. These studies found that within 1, 2, and 3 weeks of opioid-related law enforcement drug seizures, there was an increase in overdose fatalities within radii of 100, 250, and 500 meters, respectively, of the drug seizures^12^ and that nonfatal overdoses exhibited a community spread process, which was exacerbated following law enforcement strategies to disrupt the unregulated drug market.^13^ Indianapolis is a sparsely populated city and the study used fairly long exposure time periods (7, 14, and 21 days) between drug seizure observation and overdose outcome determination.

Since fentanyl entered the unregulated drug supply in San Francisco, California, around 2019,^14^ overdose mortality rates have reached record highs. This has sparked increased enforcement of drug laws. In December 2021, the Mayor declared a state of emergency in the Tenderloin neighborhood of San Francisco to enable “more coordinated enforcement and disruption of illegal activities,”^15^ followed by a declaration by the newly elected District Attorney that “combatting open-air drug markets and holding drug dealers accountable [is] a top priority of her administration.”^16^ In September 2022, the District Attorney’s Office implemented a new policy to “bundle misdemeanor drug possession charges for individuals with at least 5 misdemeanor citations for public drug use.”^17^ In May 2023, the California Governor authorized the assignment of California Highway Patrol and California National Guard personnel to a new multiagency operation with the San Francisco Police Department aimed at “targeting fentanyl trafficking, disrupting the supply of the deadly drug in the city, and holding the operators of drug trafficking rings accountable.”^18^ Medications for opioid use disorder (MOUD) are available in San Francisco, including methadone, buprenorphine, and, to a lesser extent, naltrexone. While San Francisco has a treatment-on-demand policy since 2008,^19^ there are barriers to access, especially for people who do not have insurance or the ability to pay.^20^

To determine whether there is a geospatial association between law enforcement drug seizures and opioid-involved overdose mortality, we conducted a spatiotemporal study in San Francisco using secondary data from 2020 to 2023. San Francisco is of particular interest given it is the second most densely populated large city in the US^21^ and has been engaging in heightened law enforcement drug enforcement since 2021.

## Methods

This cross-sectional study followed the Strengthening the Reporting of Observational Studies in Epidemiology (STROBE) reporting guideline. The institutional review board at RTI International determined that the study was not research involving human participants as defined by the US Department of Health and Human Services.

We conducted multivariable space-time regression modeling using an endemic-epidemic framework,^13,22,23^ examining factors associated with the background rate of overdose mortality (endemic), followed by exploring how law enforcement drug seizures may precipitate future overdose events (epidemic). None of these data sources included any personal identifying information, and thus, informed consent was not required.

### Data Sources

Our outcome variables were derived from overdose mortality data collected by the Office of the Chief Medical Examiner of the San Francisco Department of Public Health (OCME). In this study, we analyzed overdose mortality data from January 1, 2020, to August 31, 2023. The OCME draws blood from every decedent and tests for a large panel of drugs. During the study period, there were 2181 deaths in San Francisco that were determined to be caused by drug toxicity. Of these, 1833 tested positive for any opioid or synthetic opioid, including heroin and fentanyl analogs; and of these, 1686 tested positive for any fentanyl analogs. Every overdose death had a time of death and an address where the decedent was found. We have 2 primary outcomes: the time and location of (1) overdose mortality involving any opioid and (2) overdose mortality involving fentanyl or any fentanyl analog.

Our main epidemic exposure variables were constructed using publicly available crime data from the San Francisco Police Department. We used data from January 1, 2020, to August 31, 2023, from the full dataset of all crimes in San Francisco during this period. Our primary drug seizure variable was any drug seizure crime event that involved any drug distribution charge. The hypothesized mechanism for the association between law enforcement drug seizures and overdose deaths was that law enforcement removes people who provide drugs from the community (by arresting people for drug distribution), thereby making people procure drugs from new sources with unpredictable drug solutions. We also conduct sensitivity analyses where we narrow the dataset to incidents resulting in drug seizures that were listed as involving any opioids (n = 1462). Every drug seizure event had a time and location of the drug seizure. As such, the 2 exposure variables were the time and location of (1) any drug seizure event and (2) any drug seizure event involving an opioid.

We also include 7 candidate endemic explanatory variables. The first is an annual time trend, which was calculated using calendar dates. The second is a COVID-19 lockdown period indicator variable; San Francisco used shelter in place orders from March 19, 2020, to January 25, 2021. Block group-level sociodemographic measures were constructed from the 2018 to 2022 American Community Survey 5-year survey estimates.^24^

We determined the block group-level population percentage of San Francisco residents that were male, Black, Hispanic (Latino/a), Asian, American Indian or Alaska Native, White, or other race and ethnicity (including Native Hawaiian and Other Pacific Islander, some other race alone, 2 or more races). Finally, we constructed the block group-level Neighborhood Deprivation Index (NDI) for San Francisco, a standardized and reproducible index that summarizes deprivation across 5 domains of socioeconomic status (education, employment, housing, occupation, and poverty).^25^ NDI is standardized with a mean (SD) of 0 (1); higher values indicate higher deprivation. Block group-level measures were appended to overdose events by spatially joining the data.

### Statistical Analyses

We considered overdose deaths within geospatial radii of 100 meters, 250 meters, and 500 meters of drug seizures, and we considered overdose deaths within 1 day, 2 days, 3 days, and 7 days of each drug seizure event. Thus, we had 12 outcomes (3 radii each at 4 time points) for each exposure measure. Our hypothesis was that the strength of the association would lessen as the radius increased and the number of days increased from the drug seizure location and event.

We used a TWINSTIM model to determine whether spatiotemporal patterns of opioid-involved overdose mortality are consistent with a community spread process and to assess the degree to which exposure to law enforcement drug seizures may be associated with future overdose events in the surrounding community. The TWINSTIM treats overdose events as resulting from a self-exciting process (past overdose events estimate future overdose events)^26^ that decays with increasing spatial and temporal distance from the focal event and ensures that the exposure (drug seizures) precedes the outcome when assessing how drug seizures enhance the community spread of overdose. The rate of community spread at a specific location and time point, given all overdose events, was additively decomposed into endemic and epidemic components.^13^

The endemic component included a centered time trend to determine whether the strength of the endemic component shifted during the 3.5-year study period. We included an indicator of whether the fatal opioid or fentanyl overdose event occurred during the COVID-19 shelter-in-place period. The block group-level race and ethnic composition measures were highly colinear with the NDI and were dropped from the endemic model. Similarly, the block group-level percentage of males were dropped due to lack of variation across the study area. Population density at the block group-level was log-transformed and used as an offset endemic term.

The epidemic component specified a self-exciting point process effect among overdoses and described the degree to which exposure to drug seizure disruptions are associated with future overdose events. The epidemic component was implemented across multiple space-time kernels (100 meters, 250 meters, and 500 meters; 1, 2, 3, and 7 days) to assess patterns in the spatiotemporal association between community spread of overdose and exposure to local drug supply disruptions. This is conceived as each overdose event being associated with future overdose events within these space-time kernels assuming a decay of the infection force as the spatial and temporal distance from it increased. Relative risk (RR) and 95% CIs for endemic and epidemic factors were calculated and the spatiotemporal analyses were carried out using R package “surveillance.”^26^

Statistical significance was set at *P* <.05, and all tests were 2-sided. Data were analyzed from January 2020 to September 2023. R version 4.1.1 (R Project for Statistical Computing).

## Results

**Figure 1.**
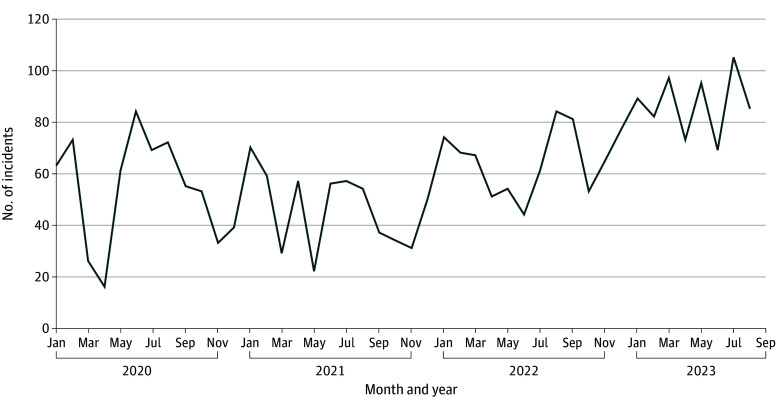
Law Enforcement Drug Seizures, January 2020 to September 2023

**Figure 2.**
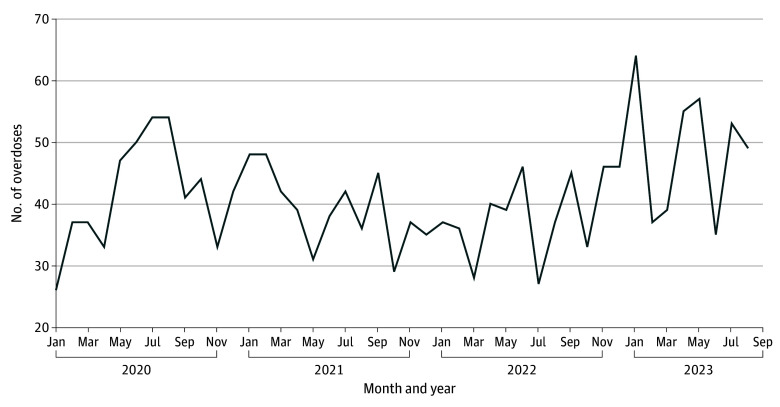
Overdoses, January 2020 to September 2023

**Figure 3.**
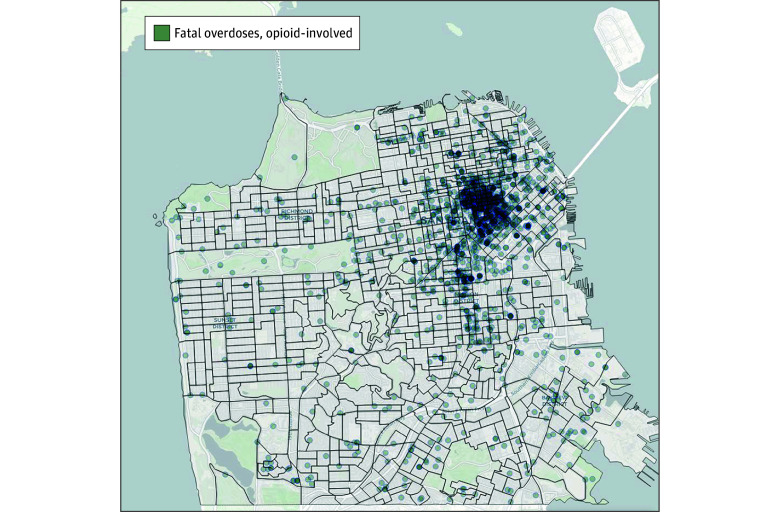
Map of Opioid-Involved Overdose Deaths in San Francisco, California, 2020 to 2023 Map was created using OpenStreetMap.^27^

During the study period, 2674 drug seizure crime events occurred, and the number of law enforcement drug seizures fluctuated monthly between 16 and 106, with the lowest dip occurring in April 2020 during the initial COVID-19 shelter-in-place ordinance (Figure 1). Drug seizures started to increase in January 2022, concomitant with the San Francisco Mayor declaring a state of emergency. Opioid-involved overdose deaths ranged from 25 to 59 per month (Figure 2). Most opioid-involved overdose deaths were located in the Tenderloin and adjacent South of Market neighborhoods (Figure 3). Of the 1833 opioid-involved overdose deaths, 713 deaths (37.9%) were exposed to law enforcement drug seizures. Of the 1686 fentanyl-involved overdoses, 665 overdoses (39.4%) were exposed to law enforcement drug seizures.

### Endemic Model

The annual time trend increased significantly for fatal opioid overdoses (RR, 107; 95% CI, 1.01-1.13; *P* = .02) and did not increase significantly for fatal fentanyl overdoses (RR, 1.05; 95% CI, 1.00-1.11; *P* = .07) (Table 1). During the COVID-19 shelter-in-place period, the rate of fatal opioid overdoses increased significantly by 31% (RR, 1.31; 95% CI, 1.14-1.50; *P* <.001). Neighborhoods with higher NDI were associated with significantly higher rates of fatal opioid overdose (RR, 1.74; 95% CI, 1.67-1.80; *P* <.001) and fatal fentanyl overdose (RR, 1.74; 95% CI, 1.67-1.80; *P* <.001) (Table 1).

**Table 1.** Risk Ratios of Endemic Variables With Opioid Overdose Mortality Outcomes, San Francisco, California, 2020-2023

Endemic variable	Any opioid, (n = 1833)		Fentanyl analog, (n = 1686)	
	RR (95% CI)	*P* value	RR (95% CI)	*P* value
Annual time trend	1.07 (1.01-1.13)	.02	1.05 (1.00-1.11)	.07
COVID-19 lockdown period	1.31 (1.14-1.50)	<.001	1.24 (1.07-1.43)	.003
Neighborhood Deprivation Index	1.74 (1.67-1.80)	<.001	1.74 (1.67-1.80)	<.001

Abbreviation: RR, relative risk.

### Epidemic Model

Exposure to a law enforcement drug seizure was significantly associated with the epidemic probability of fatal opioid overdose and fatal fentanyl overdose, with strong and consistent spatiotemporal patterning (Table 2). Within the surrounding 100 meters, drug seizures were associated with an increased risk of fatal overdoses the day following the drug seizure event (RR, 1.74; 95% CI, 1.06-2.83; *P* = .03), and the elevated risk persisted for 7 days, although the magnitude diminished over time (2 days: RR, 1.55; 95% CI, 1.09-2.21; *P* = .02; 3 days: RR, 1.45; 95% CI, 1.08-1.93; *P* = .01; 7 days: RR, 1.27; 95% CI, 1.11-1.46; *P* < .001).

**Table 2.** Risk Ratios of Police Drug Distribution Charge Seizure With Opioid Overdose Mortality Outcomes by Area and Time, San Francisco, California, 2020-2023

Seizure by area and day	Any opioid, (n = 1833)		Fentanyl analog, (n = 1686)	
	RR (95% CI)	*P* value	RR (95% CI)	*P* value
100 m				
Day 1	1.74 (1.06-2.83)	.03	NA^a^	NA^a^
Day 2	1.55 (1.09-2.21)	.02	1.47 (0.87-2.50)	.15
Day 3	1.45 (1.08-1.93)	.01	1.25 (0.81-1.93)	.31
Day 7	1.27 (1.11-1.46)	.001	1.20 (0.96-1.49)	.12
250 m				
Day 1	1.39 (1.15-1.68)	.001	1.42 (1.08-1.86)	.01
Day 2	1.36 (1.24-1.49)	<.001	1.40 (1.20-1.62)	<.001
Day 3	1.30 (1.20-1.39)	<.001	1.30 (1.16-1.45)	<.001
Day 7	1.18 (1.14-1.22)	<.001	1.18 (1.13-1.23)	<.001
500 m				
Day 1	1.29 (1.20-1.39)	<.001	1.29 (1.17-1.43)	<.001
Day 2	1.24 (1.19-1.30)	<.001	1.22 (1.16-1.29)	<.001
Day 3	1.20 (1.16-1.24)	<.001	1.18 (1.14-1.22)	<.001
Day 7	1.12 (1.10-1.14)	<.001	1.12 (1.10-1.13)	<.001

Abbreviation: NA, not available; RR, relative risk.

^a^ Model did not converge due to low number of exposed fentanyl overdose mortality events.

Similar spatiotemporal patterns were observed in the 250-meter and 500-meter spatial bandwidths. Sensitivity analyses demonstrated no substantive differences in spatiotemporal patterns for fatal fentanyl overdoses. While the effect sizes were similar for fatal fentanyl overdoses in the 100-meter bandwidth, those were not statistically significant and the model for 1 day and 100 meter did not converge due to a low number of exposed fentanyl overdose mortality events (Table 2).

## Discussion

We found that exposure to law enforcement drug seizure events was significantly associated with community spread of opioid overdose events, where the rate of future overdose events was higher in the days following and in locations around law enforcement drug seizure events in San Francisco. Within each space-time kernel, the strength of the community spread process, all of which were statistically significant, dissipated the further away in time and distance from the drug seizure event, giving further credence to the finding. These results were aligned with the results from a similar study that assessed the community spread of nonfatal overdose events following law enforcement drug seizures in Indianapolis, Indiana.^13^ That study, conducted in a sparsely populated city and with fairly long time periods (7 days and 14 days) between drug seizure observation and overdose outcome determination, found that the rate of a future nonfatal overdose was more than twice as high in close spatiotemporal proximity following an opioid-related drug seizure.

The current study was important in part because the data were from San Francisco, which is geographically small (7 × 7 miles), is the second most densely populated city in the US, and enacted a series of new law enforcement efforts to disrupt drug sales during the study period.^24,28^ The enforcement of drug laws intended to increase public safety for the residents in San Francisco may be having an unintended negative consequence of increasing risk of overdose mortality. It is possible that alternative forms of policing, such as harm reduction policing,^29^ focused deterrence,^30^ and health professional-law enforcement partnerships,^31^ could reduce the likelihood of drug overdose mortality.

There are numerous evidence-based alternatives to law enforcement that address public safety and health concerning opioid use. The first is to provide easier access to MOUD for anyone who needs it.^32^ Another approach is to provide people dependent upon opioids with a safer supply of regulated opioids.^33,34,35^ Randomized clinical trials have shown that providing a regulated supply of diacetylmorphine (the active ingredient in heroin) to people dependent upon opioids is a more effective way to retain people in treatment and to reduce their involvement in illicit drug use and other criminal activities than standard methadone treatment.^28^ A different approach adopted by some countries, including Portugal and the US state of Oregon (temporarily), has been decriminalizing possession and use of drugs. This brought down overdose mortality and HIV rates in Portugal without raising crime rates, and early data from Oregon suggests that it did not produce increases in other crimes.^36,37,38^ Another evidence-based alternative is to provide people with a supervised place to use drugs at overdose prevention sites, which have been shown to reduce overdose deaths and crime and social disorder in the neighborhoods in which they are placed.^39,40,41,42,43^

Our study found that overdose mortality in San Francisco was heightened during the COVID-19 shelter-in-place ordinance. During COVID-19, San Francisco rented hotel rooms for people experiencing homelessness. Isolating people in single room occupancy hotels was a good policy to limit COVID-19 transmission, but for those who were using drugs it meant there was no one there to witness and help them if they overdosed.^44^ Ways to mitigate this problem include installing overdose prevention sites in single-room occupancy hotels and providing a safe supply of drugs.^45,46^

In the endemic model of our study, we also found that geospatial areas with higher socioeconomic deprivation had higher overdose mortality rates. Several other studies in many countries have found similar results.^47,48,49,50^ The current study supports a large body of literature showing that living in neighborhood with lower socioeconomic indicators was associated with poor health outcomes. Evidence-based programs to reduce overdose mortality should focus on neighborhoods with high NDI.

### Limitations

This study has limitations. There is the potential for misclassification of our main variables. We cannot be certain that the location and time of every overdose death was correctly assessed and documented. There may also be misclassifications in location, time, and type of drug seizures made by law enforcement. However, such misclassification would likely bias the data toward null findings. Drug seizures involving opioids are likely to be undercounts because the San Francisco Police Department does not test every drug solution they seize. This also means we cannot assess whether drug solutions that are seized by law enforcement are similar to the drug solutions that are in decedents, including whether they contain more potent fentanyl analogs. There is very little information on most aspects of the unregulated drug market in US cities, which is likely the most impactful factor with respect to overdose mortality. Drug markets are not randomly located in a city, and where people overdose may not be indicative of where they purchase drugs. We do not know if those arrested for distributing drugs were actually doing so, for how long, or to how many people. Policing practices in San Francisco may not generalize to other locations or forms of policing.

## Conclusions

As we are confronted with overdose rates that have contributed to a decline in US life expectancy since 2020,^51^ we need to consider how existing policies are contributing to this significant public health problem. To our knowledge, no evidence-based approach exists that shows supply-side interventions, such as policing and seizing drugs, reduces drug use or drug-related health problems. Instead, our study suggests that law enforcement interventions to arrest people selling drugs in the unregulated drug market and seize the drugs they are selling may have deleterious outcome on public health by increasing overdose mortality. We recommend trying evidence-based approaches that have worked elsewhere, including providing a safe supply of drugs to people who are dependent, offering no-cost substance use disorder treatment on demand, drug decriminalization, and providing a safer environment to use drugs, such as overdose prevention sites.

## Notes

Supplement.
Data Sharing Statement
