# Supplementary material for: Law Enforcement Drug Seizures and Opioid-Involved Overdose Mortality
Source: JAMA Netw Open. 2025 Mar 19;8(3):e251158. doi: 10.1001/jamanetworkopen.2025.1158 (PMC11923703; doi:10.1001/jamanetworkopen.2025.1158)
Supplement: Supplement. — Data Sharing Statement [file jamanetwopen-e251158-s001.pdf]

Data Sharing Statement

Data
Data available: No

Additional Information
Explanation for why data not available: The data is already publicly available
